# Homogenization of Maxwell’s equations in a layered system beyond the static approximation

**DOI:** 10.1038/s41598-020-72727-8

**Published:** 2020-09-25

**Authors:** Alexander M. Merzlikin, Roman S. Puzko

**Affiliations:** 1 Moscow Institute of Physics and Technology, 9 Institutskiy per., Dolgoprudny, Moscow 141700 Russia; 2 Dukhov Automatics Research Institute, Sushchevskaya street 22, Moscow, 127055 Russia; 3 Institute for Theoretical and Applied Electromagnetics, Russian Academy of Sciences, 13, ul. Izhorskaya, Moscow, 125412 Russia

**Keywords:** Theory and computation, Optical physics, Optical materials and structures

## Abstract

The propagation of electromagnetic waves through a disordered layered system is considered in the paradigm of the homogenization of Maxwell’s equations. Although the accuracy of the effective dielectric permittivity and/or magnetic permeability is still unclear outside the static approximation, we show that the effective wave vector can be correctly introduced even in high-frequency cases. It is demonstrated that both the real and imaginary parts of the effective wave vector are self-averaging quantities connected by the Kramers–Kronig relations. We provide a unified approach to describe the propagation and localization of electromagnetic waves in terms of the effective wave vector. We show that the effective wave vector plays the same role in describing composite materials in electrodynamics as the effective dielectric permittivity does in statics.

## Introduction

**Figure 1:**
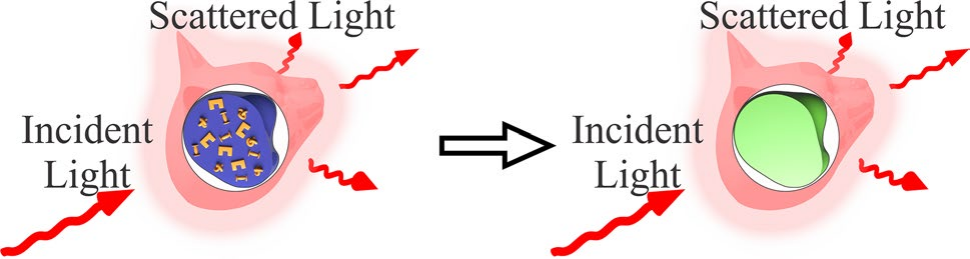
Homogenization problem.

The electrodynamic properties of artificial composite materials depend on their structure and the component compounds and can differ substantially from the properties of homogeneous materials^1^. Nevertheless, the description of metamaterials as homogeneous by means of their effective parameters (dielectric permittivity and magnetic permeability, chirality coefficients, etc.) remains a relevant problem in electrodynamics. The problem of replacing an inhomogeneous composite system with a homogeneous material with effective parameters is called the homogenization problem (detailed formulation of the problem is given in the literature^2^). It is assumed that the effective parameters of a homogeneous system provide the same scattered field as that of an inhomogeneous system (see Fig. 1). That is, the effective parameters allow the description of light scattering by a composite system without considering the inhomogeneous structure of the system.

The accuracy of the homogenization problem is under active study in connection with the characteristic scales^3^—system length *L*, inhomogeneity size $\xi$ and wavelength $\lambda$. As a rule, it is assumed that the linear size *L* of the composite system is sufficiently large and the characteristic size of the inhomogeneities is sufficiently small that the composite can be considered a homogeneous material in the macroscopic sense. In addition, if we have a procedure that provides the effective parameters for a finite sample of size *L*, then as *L* increases, these parameters should tend to some constant values (this property is called “self-averaging”).

Notably, the homogenization problem in electrostatics differs substantially from the one in electrodynamics. In the static case (the rigorous theory of the homogenization problem is constructed only for the static case), Maxwell’s equations are divided into two independent groups of equations—magnetic and electric. Therefore, the description of inhomogeneous media by means of effective dielectric permittivity $\varepsilon _{eff}$ and magnetic permeability $\mu _{eff}$ is quite natural. The microscopic description of each of the fields (electric and magnetic) reduces to a dimensionless Laplace equation, and a scaling approach can be applied^4^. Currently, the greatest progress has been made in homogenizing Maxwell’s equations for the static case. Namely, the scaling theory of G-convergence^4^ and spectral theory^5–8^ have been developed.

In contrast to statics, the electric and magnetic fields are related to each other in the case of dynamics. In particular, the amplitudes of the electric and magnetic fields of the plane wave are connected by admittance *Y*. In addition, the homogenization problem in dynamics is multiscale—there are additional scales associated with the equations, namely, the set of local wavelengths $\lambda /\sqrt{\varepsilon \mu }$, where $\lambda =2\pi /k_0$ is the wavelength in vacuum and $k_0=\omega /c$ is the wavenumber. The presence of multiple scales leads to difficulties in the transition from a finite system to an infinite system^9^, and the results of the G-convergence theory cannot be applied in the general case. Thus, two questions arise when the theory of homogenization for dynamics is being constructed: which parameters should be chosen as effective ($\varepsilon _{eff}$, $\mu _{eff}$ or $n_{eff}$, $Y_{eff}$ or some others), and how should the homogenization procedure be implemented^2,10–13^?

For clarity, let us distinguish two separate approximations widely used in homogenization theory: the quasistatic approximation and the long wavelength approximation. The quasistatic approximation considers the homogenization of Maxwell’s equations in electrostatics. However, the static response (e.g., polarization) of each inhomogeneity is replaced by the response at non-zero frequency. For example, the quasistatic method adopts the solution of the static homogenization problem for a one-dimensional system, which has been known for more than a century. However, the approach is invalid with respect to metamaterial description: resonant responses of inhomogeneities (Mie resonance, split-ring resonances, etc.) exist in the case of the temporal differential equation only. That is, the radiation illuminated by inhomogeneities should be calculated in the framework of a time-dependent problem. The second approximation is the long wavelength approximation, which considers the solution of Maxwell’s equations in terms of $\xi /\lambda$ expansion, where $\xi$ is the inhomogeneity size and $\lambda$ is the wavelength. However, *ab initio* simulations of light scattering in layered systems^14^ indicate a disagreement between the numeric experiment and the predictions of long wavelength approximation theory in the first order of $\xi /\lambda$.

One of the approaches to the homogenization problem in the long wavelength approximation was proposed for periodic systems in the works of Wood et al.^15,16^ and was further developed in^17–19^. The basic idea is to determine the effective dielectric permittivity of an inhomogeneous periodic dielectric medium by means of a Bloch wave vector $k_{Bl}=k_0\sqrt{\varepsilon _{eff}}$. It is implied that in the absence of magnetic components, we can assume $k_{Bl}=k_0\sqrt{\varepsilon _{eff}}$. In essence, this approach is equivalent to the fact that we transfer the independence of the electric and magnetic fields from statics to dynamics (that is, the effective permittivity and permeability are introduced independently by the homogenization procedure). Specifically, the impedance is implied to be equal to the inverse refractive index. This is a common case in the optics of natural media. However, this approach inaccurately describes metamaterials with artificial magnetism.

An alternative approach proposed by Rytov and Levin^20–26^ for layered systems was generalized to two-dimensional and three-dimensional cases^27–33^. The basic idea is the averaging of the fields over the unit cell (or over its surface^13^) of the periodic medium.

Two quantities are defined separately for the periodic system within Rytov’s approach: the effective wave vector $k_{eff}^{Ryt}=\sqrt{\varepsilon _{eff}^{Ryt}\mu _{eff}^{Ryt}}k_0$ and the effective admittance $Y_{eff}^{Ryt}=\sqrt{\varepsilon _{eff}^{Ryt}/\mu _{eff}^{Ryt}}$. The effective wave vector is set equal to the Bloch wave vector $k_{Bl}$. The effective admittance is obtained by field averaging over the unit cell volume: $Y_{eff}^{Ryt}=\langle H \rangle /\langle E \rangle$. However, this approach leads to a somewhat dubious result: either $\varepsilon _{eff}$ or $\mu _{eff}$ has a negative imaginary part. Moreover, the introduced effective parameters fail validation by direct calculation of wave propagation through the finite system. The parameters calculated from scattering data by means of the R–T retrieval approach^34–36^ oscillate with the system length, i.e., $\varepsilon _{eff}$ and $\mu _{eff}$ are not self-averaging quantities^14,37^. The introduction of the parameters $\varepsilon _{eff}$ and $\mu _{eff}$ might be incorrect for layered systems outside the static approximation^37–39^.

In summary, the theory of the homogenization problem beyond the static case is still under intense investigation.

Recently, we have shown^40^ that the effective refractive index of a periodic system self-averages not only in the long wavelength approximation but also in high-frequency cases. However, periodic systems differ from disordered systems. Specifically, Anderson localization of light occurs in the latter.

In this paper we consider the normal incidence of light on the layered structure. The effective wave vector has only one non-zero component, which is parallel to the system axis. We refer to that component as the wave vector. We focus our attention on the properties of the effective wave vector $k_{eff}$ in the general case of disordered systems. We are interested in the self-averaging property of the effective wave vector defined by the transmission approach^40^. The self-averaging property of $k_{eff}$ is critical for the homogenization problem. The studies are performed by the numerical computation in different frequency domains and even in the high-frequency case. In addition, we generalize the Kramers–Kronig-like relations known for the periodic systems^40^ to the case of the disordered systems, which is important for establishing the analytical relation between the localization length and the real part of the effective wave vector.

## Results

### Self-averaging of the effective wave vector

We study wave propagation through a disordered system composed of dielectric layers with different dielectric permittivities. For simplicity, we assume that all the layers have the same thickness.

The standard approach to the localization and propagation of light considers a disordered system immersed in a vacuum. In this case, the transmission and reflection coefficients and other scattering data are affected by both the Anderson localization^41^ and the effect of reflection from boundaries between the vacuum and the disordered system. To separate the localization effect from the scattering at the boundaries, we immerse the system into a medium with an averaged dielectric permittivity (the averaging is taken over the permittivity probability distribution).

The transmission coefficient of a one-dimensional disordered system can be represented in the following form:

1 $$\begin{aligned} t=|t|\mathrm {exp}\left( i\phi \right) =\mathrm {exp}\left( i\mathrm {Im}(\ln t)+\ln {|t|}\right) . \end{aligned}$$

The propagation of an electromagnetic wave through the inhomogeneous layer can be viewed as a three-stage process: transmission of the external medium-layer boundary, propagation through the volume of the layer, and transmission of the layer-external medium boundary.

The homogenization problem reduces to two independent problems from that perspective: determining the effective wave vector and considering the boundary conditions. To solve the problem of boundary conditions, regularization of the effective impedance by means of additional surface currents^42^ or an additional effective layer at the boundary^43^ was previously proposed. The problem of boundary conditions is also considered in the profound variation of Rytov’s approach^13^, where the finite samples are studied. In the latter case, the field averaging is conducted over the unit cell, which is far from the system boundaries, to avoid boundary effects. In this paper, we shift our attention away from the boundary problem and focus on the effective wave vector.

Notably, the contribution of the boundaries of a system to the transmission coefficient does not increase (or increases more slowly than exponentially) as the system thickness increases. At the same time, propagation through the layer is characterized by an effective wave vector, whose contribution to the phase or exponential decay (associated with absorption or scattering of light) of the transmission coefficient increases linearly with the thickness *L* of the system. Thus, the reasonable approximation used in our discussion neglects the surface effects for large *L* (that is, the phase is much larger than $\pi$, i.e., $L\gg \lambda$). Therefore, the imaginary part of the logarithm of the transmission coefficient $i\mathrm {Im}({\ln }t)$ is a phase accumulated along the thickness *L* of the system. The immersion of the sample in a medium with an averaged permittivity reduces the reflections from the boundaries of the sample. It is helpful in the case of long wavelengths where the reduction of boundary contributions allows consideration of smaller systems (compared to the vacuum case).

Note that the phase $\phi$ of the transmission coefficient (1) is a multivalued function. The single branch of the function should be chosen for later discussion. We consider two procedures of phase restoration: “by frequency” and “by propagation”. The phase restoration by frequency (by propagation) is based on the assumption that the phase $\phi$ is a continuous and monotonically increasing function of the frequency (coordinate). Both these procedures lead to the same value of the phase and are used in this paper. For convenience, we refer to the phase restored by frequency (or by propagation) as phase, and the phase reduced to the region $[0,2\pi ]$, as reduced phase.

The real part of ${\ln }|t|/L$ describes the decay of the wave amplitude, which is determined by the imaginary part of the wave vector. Thus, the effective wave vector for a thick system can be defined as follows^40^:

2 $$\begin{aligned} \mathrm {Re}k_{eff}= & {} \frac{\mathrm {Im}({\ln }t)}{L}, \end{aligned}$$

3 $$\begin{aligned} \mathrm {Im}k_{eff}= & {} -\frac{\mathrm {Re}({\ln }t)}{L}. \end{aligned}$$

These formulas can be combined into

4 $$\begin{aligned} k_{eff}=-i\frac{{\ln }t}{L}. \end{aligned}$$

The definition of $k_{eff}$ by means of the transmission coefficient is valid for periodic systems as confirmed by numerical calculations^14,40^.

**Figure 2:**
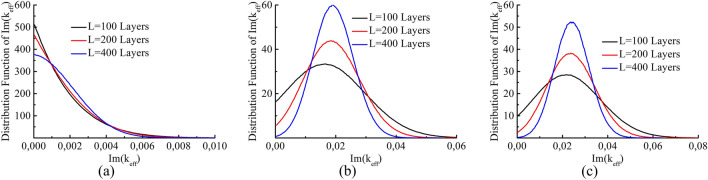
Distribution functions of $\text {Im}(k_{eff})$ for random systems composed of 100 (black), 200 (red) and 400 (blue) layers. The dielectric permittivity of the layers $\varepsilon$ is uniformly distributed over the interval [1; 11] and the dielectric permittivity of the external medium is $\langle \varepsilon \rangle$. The thickness of each layer is (**a**) $k_0d=0.1$; (**b**) $k_0d=1.0$; (**c**) $k_0d=10.0$. The ensembles used in the calculations consist of $10^7$ realizations.

By definition (3), the imaginary part of the wave vector converges to the inverse length of the localization:

5 $$\begin{aligned} \gamma =\frac{1}{L_{loc}}=\lim _{L\rightarrow \infty }\frac{-\text {ln}|t|}{L}=\lim _{L\rightarrow \infty }(\text {Im}k_{eff}). \end{aligned}$$

where $L_{loc}$ is the localization length. The inverse length of localization $\gamma$ shows the rate of exponential decay of localized state in the disordered system. The quantity $\gamma$ is a self-averaging quantity called the Lyapunov exponent in the theory of Anderson localization^44^. That is, the imaginary part of the wave vector self-averages as the system length increases. That is also confirmed by the numerical calculations (see Fig. 2). The existence of the Anderson localization scale—the Anderson localization length—is equivalent to the self-averaging of $\mathrm {Im}k_{eff}$.

**Figure 3:**
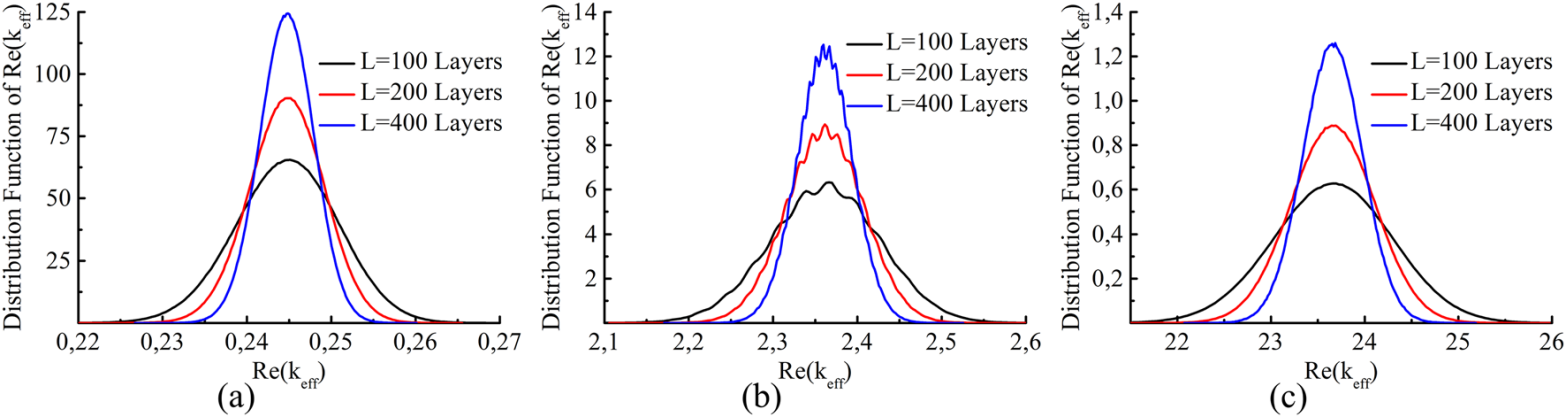
Distribution functions of $\text {Re}(k_{eff})$ for random systems composed of 100 (black), 200 (red) and 400 (blue) layers. The parameters are the same as those in Fig. 2. The thickness of each layer is (**a**) $k_0d=0.1$; (**b**) $k_0d=1.0$; (**c**) $k_0d=10.0$.

Let us now consider the real part of the effective wave vector. Calculations show that the real part demonstrates similar behavior (Fig. 3): its distribution becomes narrower as the system length increases.

**Figure 4:**
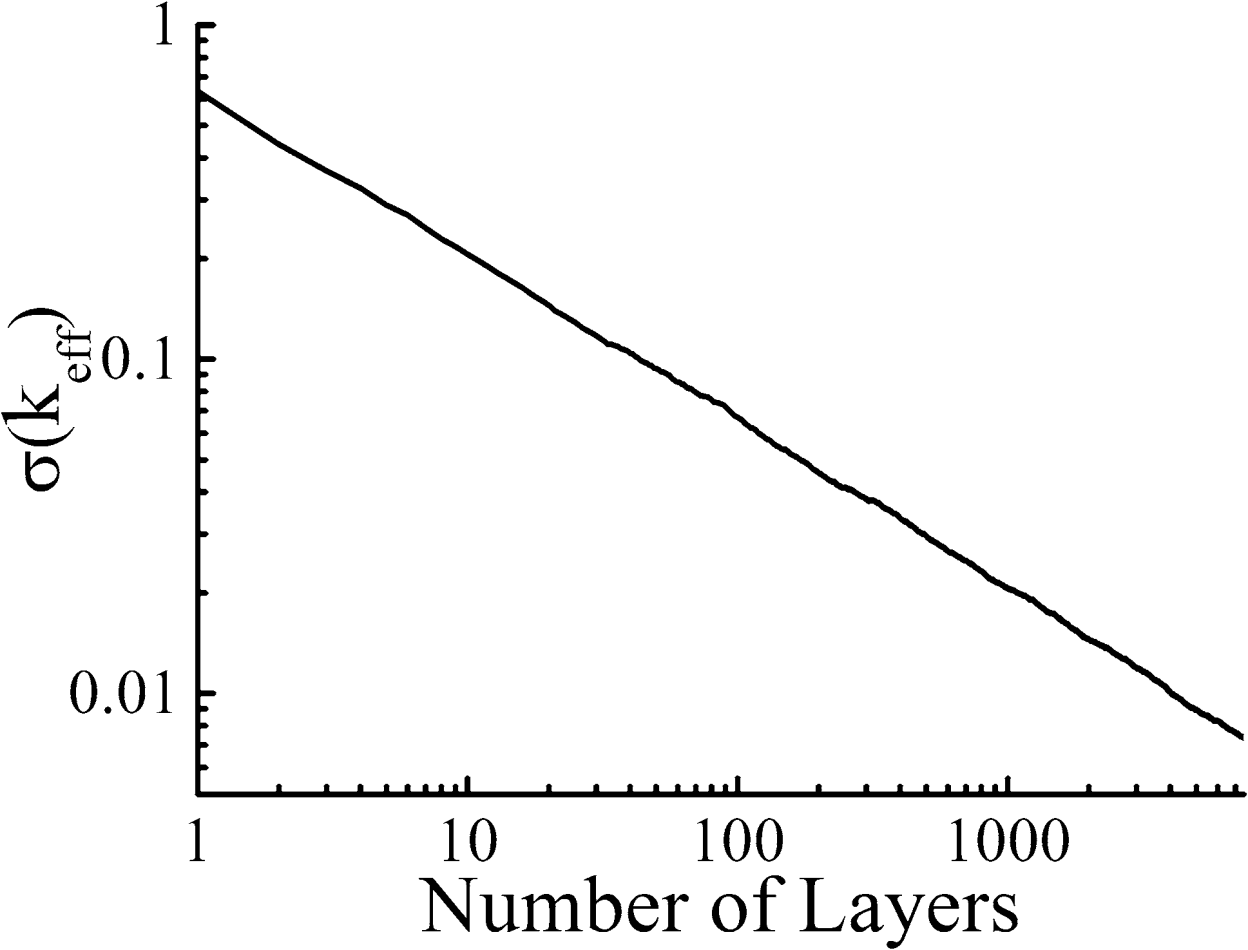
The dependency of the variance of the $\mathrm {Re}(k_{eff})$ distribution on the number of layers. The dielectric permittivity of the layers is uniformly distributed over the interval [1; 11]. The thickness of each layer is $k_0d=1$. The ensembles used in the calculations consist of $10^3$ realizations.

As seen from the graphs in Figs. 2 and 3 for the real and imaginary parts of the effective wave vector, self-averaging occurs as the system thickness increases, i.e., the distribution function becomes narrower. The rate of self-averaging can be estimated. The variance of the distribution function as a thickness function is shown in Fig. 4. The dependency of the variance on the system length follows a power law $\sigma \left( k_{eff}\right) \sim L^{-\alpha }$. The exponent obtained from the calculations is $\alpha \approx {0.5}$. Thus, the effective wave vector is expected to reach a certain value for a sufficiently long system. In other words, both the imaginary and the real parts of the effective wave vector self-average.

Thus, we have shown numerically that $k_{eff}$ determined by formula (4) self-averages as the thickness of the system increases not only in the long wavelength approximation but even outside of this approximation. Moreover, convergence occurs for the high-frequency cases when the wavelength is on the order of the layer thickness or even significantly smaller. At the same time, the effective wave vector acquires an imaginary part due to wave localization in the system. Notably, the number of layers is important for self-averaging (quantity averaging over the few layers is unlikely). However, self-averaging occurs even for high-frequency cases for sufficiently large (composed of many layers) systems.

Now, let us compare definition (4) with other definitions of the effective wave vector. Numerical calculations for an ensemble of disordered systems show that in the long wavelength approximation the value of the effective wave vector calculated by formula (2) coincides with that obtained from R–T retrieval^34^. The value of $k_{eff}$ averaged over the ensemble in the long wavelength limit for both definitions are close to the function $k_{st}=\sqrt{\langle \varepsilon \rangle }k_0$ (which corresponds to effective permittivity in the static case $\varepsilon _{st}=\langle \varepsilon \rangle$), where the dielectric permittivity is averaged over the distribution. Therefore, we assumed in the calculations that the admittance of the external medium is $Y_{av}=\sqrt{\langle \varepsilon \rangle }$. Utilizing an external medium with this value of admittance makes it possible to reduce reflections from the boundaries of the system and, thus, reduce the dependency of the transmission coefficient on the boundaries. At the same time, the introduction of an admittance differing from that of vacuum is not essential if the size of the system is substantially larger than the wavelength.

The definition of the effective wave vector was introduced earlier in the literature^45^. The multilayered system is considered as a unit cell of an infinite photonic crystal. According to the approach^45^ the effective wave vector is equal to wave vector $k_{Bl}$ of the Bloch wave in the photonic crystal. The definition was applied^45^ to analyze the Anderson localization in layered systems. Specifically, it was shown that $k_{Bl}$ gains the non-zero imaginary part for almost all frequencies if the system length increases. Moreover, the imaginary part of $k_{Bl}$ tends to the Lyapunov exponent (5) if the system length increases, i.e., $\langle {\mathrm {Im}k_{Bl}}\rangle \xrightarrow [L\rightarrow {\infty }]{}\langle {\mathrm {Im}k_{t}}\rangle$ for almost all frequencies, where $k_t$ is the effective wave vector (4). Let us now consider the convergence $\langle {\mathrm {Re}k_{Bl}}\rangle \xrightarrow [L\rightarrow {\infty }]{}\langle {\mathrm {Re}k_{t}}\rangle$.

Numerical calculations show that the difference $(k_t-k_{Bl})$ between the two definitions of the effective wave vector decreases as the system size increases. Notably, the real part of $k_{Bl}$ frequently changes with the thickness of the system by jumps of size $\sim \pi /L$. This occurs because the Bloch wave vector corresponds to the band gap for almost all frequencies for sufficiently long system^45^. Although the phase shift on the period of the considered photonic crystal changes abruptly, $k_{Bl}$ and $k_t$ tend to the same value.

Thus, the effective wave vector introduced by (4) corresponds to the definitions given in literature^34,45^.

### Qualitative arguments on the self-averaging of the effective wave vector

Following Sheng^46^, let us consider two large (consisting of many layers and with a total thickness considerably larger than the wavelength) pieces of one disordered system referred to as pieces A and B. The transmission coefficients of A and B are $t_A$ and $t_B$, respectively. The system composed of these two pieces has transmission coefficient $t_{AB}$, which equals (in order of magnitude) $t_{AB}\approx {t_A}{t_B}$. Now, adding one more piece, referred to as C, we assume that $t_{ABC}\approx {t_{AB}t_C}\approx {t_A}{t_B}{t_C}$, with logarithmic accuracy

6 $$\begin{aligned} \ln {t_{ABC}}\approx \ln {t_A}+\ln {t_B}+\ln {t_C} \end{aligned}$$

This relation is valid for both modules and phases but only if we choose the phases reconstructed by propagation, that is, the phase of the wave is supposed to be an increasing function of system thickness.

Since $\ln {t_i}$ are independent and identically distributed, $\frac{1}{N}\sum _{i=1}^{N}\ln {t_i}$ is asymptotically Gaussian distributed in accordance with the law of large numbers. The variance of the distribution is $\sigma \sim {1/\sqrt{N}}$, i.e., $D=\sigma ^2\sim {1/L}$ when all the layers of the system have the same thickness. Therefore, the quantities $\phi /L$ and $\gamma$ are self-averaging; moreover, $D_\gamma \sim {D_k}\sim 1/L$, which corresponds to the numerical experiment (see Fig. 4).

### Frequency dependence of the real and imaginary parts on the effective wave vector

**Figure 5:**
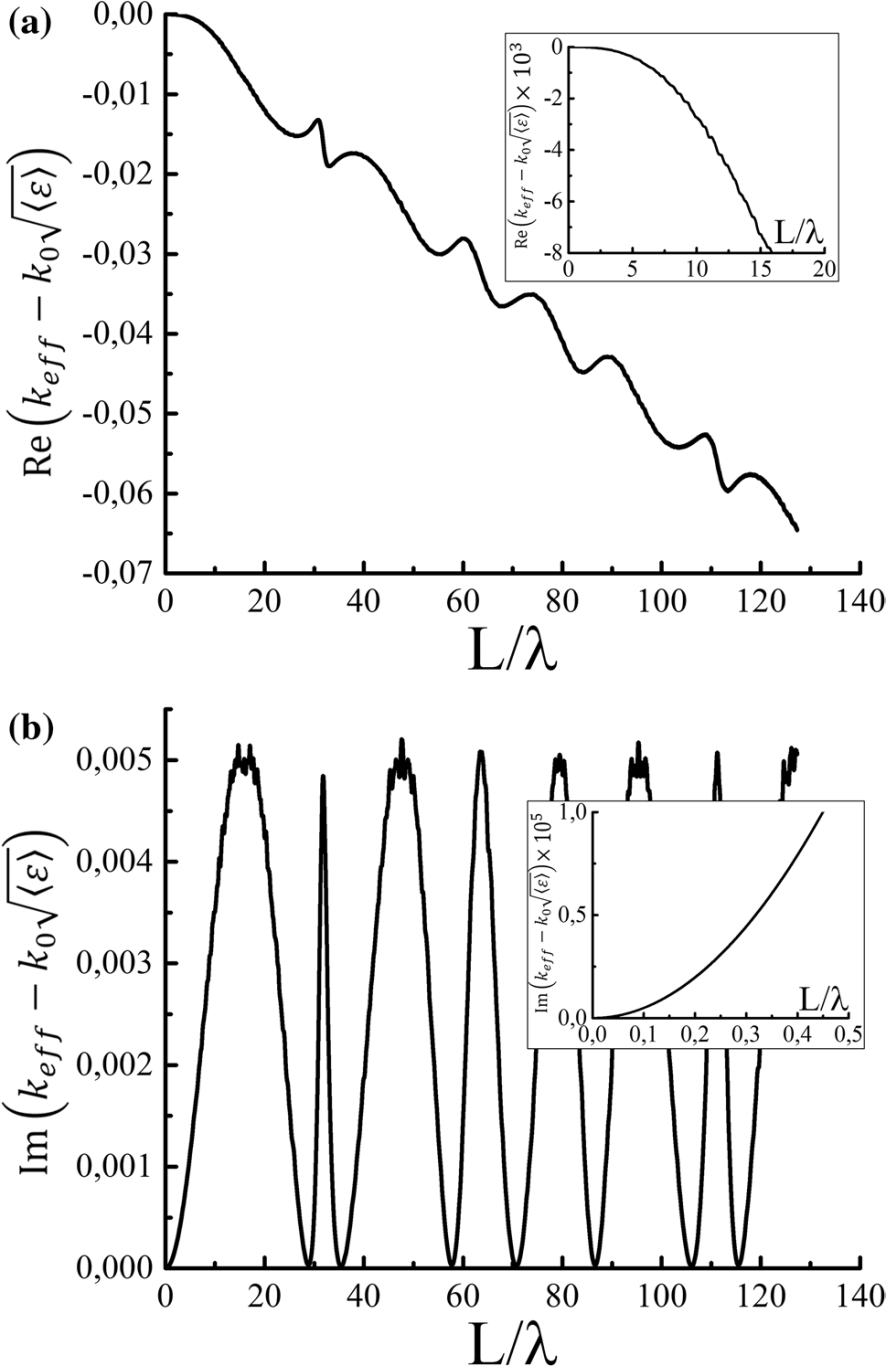
Frequency dependencies of the (**a**) real and (**b**) imaginary parts of $k_{eff}-k_0\sqrt{\langle \varepsilon \rangle }$ averaged over ensemble of random systems. Each system is composed of 100 layers. The ensemble consists of 40,000 realizations. The dielectric permittivity of each layer is taken to be $\varepsilon _1=2$ or $\varepsilon _2=3$ with equal probability.

Consider the dispersion properties of the introduced effective wave vector. The graphs of the dependencies are shown in Fig. 5 for layered systems composed of two types of layers. The effective wave vector is close to the value $k_{st}=\sqrt{\langle \varepsilon \rangle }k_0$ in the long wavelength limit. However, as the frequency increases, the dependency changes and becomes $k_{eff}\approx \langle \sqrt{\varepsilon }\rangle {k_0}$. The imaginary part of $k_{eff}$ becomes 0 at some points because one of the layers becomes transparent at these frequencies, i.e., the transmission coefficient of the layer equals $\pm 1$. The dispersion dependency of the imaginary part of $k_{eff}$ behaves as $\mathrm {Im}k_{eff}\sim \lambda ^{-2}$ in the long wavelength regime (see the inset in Fig. 5b), which is in accordance with the known behavior of the Lyapunov exponent.

### Kramers–Kronig-like relations for the effective wave vector

Since both $k=\mathrm {Re}k_{eff}$ and $\gamma =\mathrm {Im}k_{eff}$ are self-averaging quantities, a possible connection between them and their physical meaning are of interest.

We examine this question by means of the transfer matrix method^47^ (see “Methods” section). Clearly, the transmission coefficient of the layered system is an analytic function of the frequency^40^. The T-matrix of the whole system can be written in terms of the transmission and reflection coefficients as follows

7 $$\begin{aligned} T=\begin{pmatrix} t-\frac{r_Lr_R}{t} &{} -\frac{r_L}{t}\\ \frac{r_R}{t} &{} \frac{1}{t} \end{pmatrix} \end{aligned}$$

where *t* is the transmission coefficient (the transmission coefficient is independent of the direction), $r_L$ and $r_R$ are reflection coefficients for the left and right sides of the system, respectively. On the other hand, the T-matrix of the whole system is the product of the T-matrices of the homogeneous layers forming the system. All elements of these T-matrices are analytic functions of the frequency. Thus, the transmission coefficient, being a fractionally rational function of these elements, is also an analytic function of the frequency. Consequently, the function

8 $$\begin{aligned} \frac{1}{t}\frac{dt}{d\omega }=\frac{d\ln (t)}{d\omega }=\frac{dk_{eff}}{d\omega } \end{aligned}$$

should also be analytical. Therefore, the following relations are valid:

9 $$\begin{aligned} \frac{d\mathrm {Re}k_{eff}(\omega )}{d\omega }= & {} const+\frac{1}{\pi }v\cdot p\cdot \int _{-\infty }^{+\infty }\frac{\frac{d\mathrm {Im}k_{eff}(u)}{du}}{u-\omega }du, \end{aligned}$$

10 $$\begin{aligned} \frac{d\mathrm {Im}k_{eff}(\omega )}{d\omega }= & {} -\frac{1}{\pi }v\cdot p\cdot \int _{-\infty }^{+\infty }\frac{\frac{d\mathrm {Re}k_{eff}(u)}{du}}{u-\omega }du. \end{aligned}$$

The Kramers–Kronig-like relations are well-known properties of optical parameters connected to the causality principle^48^. The Kramers–Kronig-like relations (9) and (10) show that the effective wave vector being the effective parameter of the disordered system still satisfies the essential principle of causality.

The relations (9) and (10) are the important ones to reveal the physical meaning of the introduced effective wave vector. With respect to the definition of the Lyapunov exponent $\gamma (\omega )$, the second relation can be rewritten as (we make the notation $k=\mathrm {Re}k_{eff}$)

11 $$\begin{aligned} \frac{d\gamma (\omega )}{d\omega }=-\frac{1}{\pi }v\cdot p\cdot \int _{-\infty }^{+\infty }\frac{\frac{dk(u)}{du}}{u-\omega }du \end{aligned}$$

Taking into account parity $k(-\omega )=-k(\omega )$ (hence, $dk/d\omega$ is an even function of frequency) and utilizing the relation between the wave vector and the density of states ($\rho (\omega )=dk/d\omega$), one can obtain

12 $$\begin{aligned} \frac{d\gamma (\omega )}{d\omega }=-\frac{2}{\pi }v\cdot p\cdot \int _{0}^{+\infty }\frac{\omega \rho (u)}{u^2-\omega ^2}du \end{aligned}$$

Performing integration over the frequency, one obtains the Jones–Herbert–Thouless formula^49,50^

13 $$\begin{aligned} \gamma (\omega )=\frac{1}{\pi }v\cdot p\cdot \int _{0}^{+\infty }\rho (u)\ln \left| 1-\frac{\omega ^2}{u^2}\right| du \end{aligned}$$

Thus, the frequency derivative of the real part of the effective wave vector corresponds to the density of states, which confirms that $k_{eff}$ has physical grounds to be considered an effective wave vector.

### Phase randomization

It was shown in the preceding sections that the effective wave vector self-averages. Let us now consider in detail the behavior of phase $\phi$ (1), which is directly related to the effective wave vector for each system realization as $\phi =-i\ln \left( t/|t|\right) =\mathrm {Im}\ln {t}=k_{eff}L$. Although $k_{eff}$ self-averages and is consequently a deterministic quantity, the phase randomizes; more precisely, the reduced phase, which is a phase reduced to an interval $[0,2\pi ]$, randomizes. Phase randomization is used as the basis for developing a random T-matrix approach^51–54^.

The apparent contradiction can easily be explained by noting that the variance of the wave vector tends to zero as $D_k=\sigma _k^2\sim 1/L$ and the variance of the phase increases as $D_\phi =L^2\sigma _k^2\sim {L}$. Since the distribution of $k_{eff}$ is similar to the Gaussian distribution (see Fig. 3a), the distribution for the total phase is expected to be similar to the Gaussian one, but as the system thickness increases, the distribution shifts and becomes wider.

**Figure 6:**
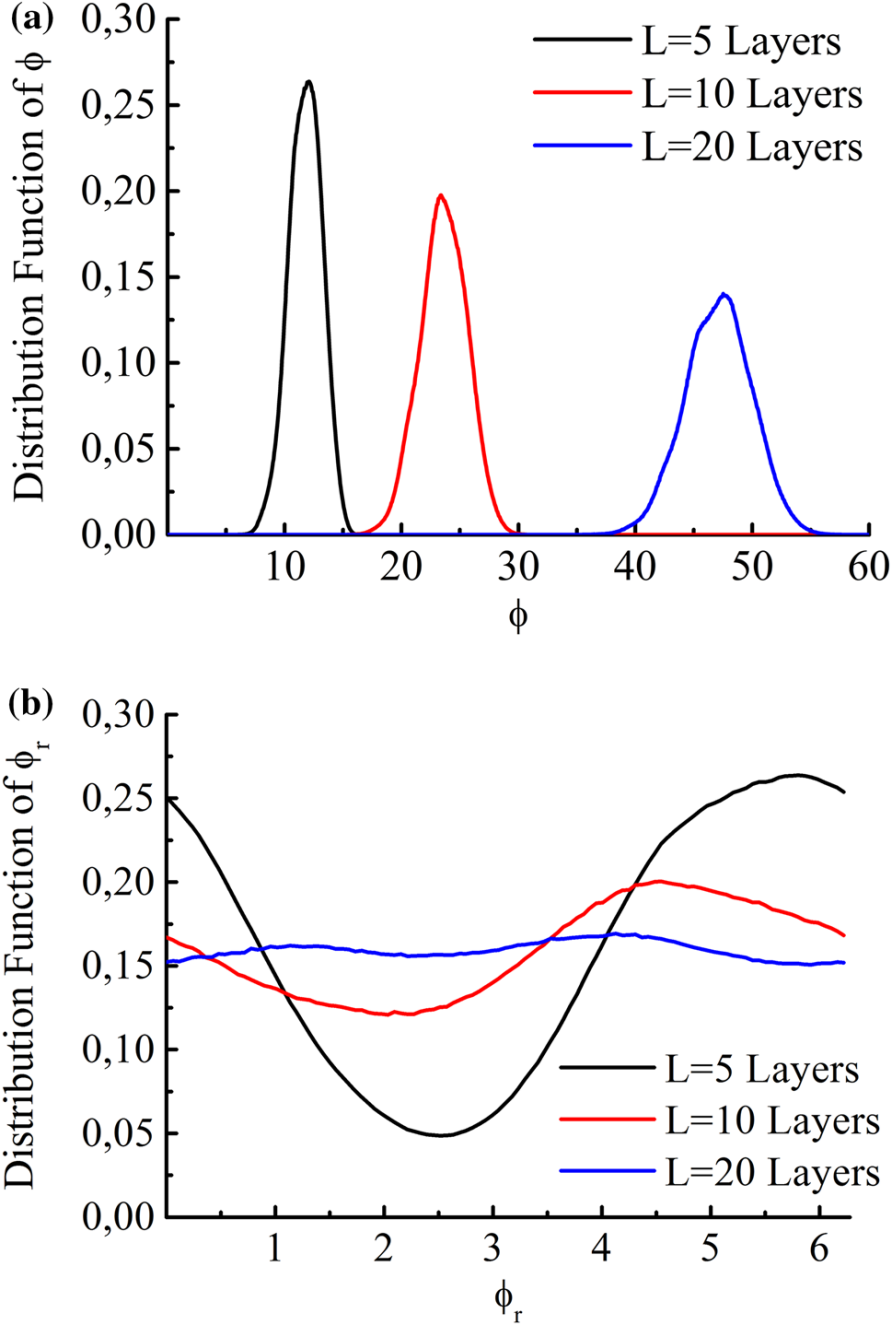
Distribution functions of (**a**) phase $\phi$ and (**b**) reduced phase $\phi _r$ for random systems composed of 5 (black), 10 (red) and 20 (blue) layers. The parameters of the layers and the external media are the same as those in Fig. 2.

The numerical calculations confirm this behavior (Fig. 6). The distribution shifts to the right proportionally to the system size and broadens. Therefore, after the reduction to the interval $[0,2\pi ]$, the distribution of the reduced phase should be close to a constant for a sufficiently large system (Fig. 6b).

However, even for a thick system, the calculations show that the distribution function differs from a constant due to the presence of an additional correlation scale—the width of the layer.

## Discussion

In this paper, we considered the propagation of an electromagnetic wave through a disordered layered system. The majority of approaches to the homogenization problem propose introducing corrections (which are proportional to the relation $\xi /\lambda$ between the inhomogeneity size and the wavelength) to the static effective dielectric permittivity and/or magnetic permeability. For example, the second-order theory describes chirality, magnetic dipoles and electric quadrupoles^55^. However, the theory experiences breakdown as the parameter $k_0d$ increases, and its validity depends on the system length^55^. These approaches have a limited field of application and are inaccurate even in the long wavelength approximation^37–39^: the effective parameters $\varepsilon _{eff}$ and $\mu _{eff}$ vary with the number of layers (that is, they oscillate), which leads to breakdown of the approaches. However, in this work, we showed that the effective wave vector $k_{eff}$ tends to a constant value for different relations as the number of layers increases. In other words, it self-averages and is thus a macroscopic quantity. The self-averaging of $k_{eff}$ is observed not only in the long wavelength approximation $\xi /\lambda \ll 1$ but even in the high-frequency cases of $\xi /\lambda \sim 1$ and $\xi /\lambda \gg 1$.

The introduction of disorder into periodic systems substantially changes the spectral characteristics, such as the density of states and the transmission and reflection coefficients^56,57^. Moreover, the disorder in photonic structures leads to Anderson localization^41^. Nevertheless, the complex optical properties of disordered systems fit the effective wave vector description. The imaginary part of the proposed effective wave vector equals the Lyapunov exponent (inverse localization length) in the limit of infinite system length $L\rightarrow \infty$.

To clarify the physical meaning of the real part of the introduced effective wave vector, we consider the frequency derivative of the effective wave vector. We show that this quantity is an analytical function of frequency (because it can be directly expressed in terms of the propagation coefficient) and thus satisfies the Kramers–Kronig-like relations. In this work, it was proven that these relations lead to the Jones–Herbert–Thouless relation, connecting the spectral density and the localization length of eigenstates, i.e., the real part of the introduced effective wave vector gives us the correct value of the density of states.

We show that $k_{eff}$ acts as the self-averaging parameter in electrodynamics instead of $\varepsilon _{eff}$ in statics. Homogenization of the effective wave vector enables a unified description of electromagnetic wave propagation and localization.

## Methods

The numerical results given in the article are obtained by computation of transmission coefficient of wave through the multilayered system. The propagation of plane waves through layered system is studied via the transfer matrix (T-matrix) method^47^. The T-matrix *T* of a layered system relates the amplitudes of waves propagating in different directions. Specifically, the following relation exists for the case considered in the article

14 $$\begin{aligned} \begin{pmatrix} t \\ 0 \end{pmatrix} =T \begin{pmatrix} 1 \\ r \end{pmatrix} \end{aligned}$$

where *r* and *t* are the reflection and transmission coefficients, correspondingly.

The T-matrix $T_j$ of a single layer placed into an external medium (to unify the description, it is convenient to place the layer between two adjacent imaginary layers of zero width) is as follows:

15 $$\begin{aligned} T_j=\begin{pmatrix} \mathrm {cos}\rho _j+\frac{1}{2}i\left( \frac{Y_j}{Y_{ext}}+\frac{Y_{ext}}{Y_j}\right) \mathrm {sin}\rho _j &{} \frac{i}{2}\left( \frac{Y_j}{Y_{ext}}-\frac{Y_{ext}}{Y_j}\right) \mathrm {sin}\rho _j \\ -\frac{i}{2}\left( \frac{Y_j}{Y_{ext}}-\frac{Y_{ext}}{Y_j}\right) \mathrm {sin}\rho _j &{} \mathrm {cos}\rho _j-\frac{1}{2}i\left( \frac{Y_j}{Y_{ext}}+\frac{Y_{ext}}{Y_j}\right) \mathrm {sin}\rho _j \end{pmatrix} \end{aligned}$$

where $Y_{ext}$ is the external medium admittance, $\rho _j=n_jk_0d$ is the optical thickness and $Y_j=n_j$ is the admittnace in the case of normal incidence. The T-matrix of a system composed of N layers is obtained by multiplying the T-matrices of the layers in reverse order

16 $$\begin{aligned} T=T_{N}\cdot T_{N-1}\cdot \cdots \cdot T_{2}\cdot T_{1}. \end{aligned}$$

Equation (14) enables calculation of the transmission coefficient. Notably, the calculation of the transmission coefficient phase is performed according to the “by propagation” phase recovery procedure discussed in the text. The straightforward application of Eq. (4) yields the value of the effective wave vector.

The method allows for a mathematically precise solution of Maxwell’s equations in one-dimensional geometry. Thus, the only inaccuracies in the calculations are the numerical errors due to the number precision limit. The smallness of the inaccuracies was monitored in the calculations by evaluating T-matrix invariants: $|T|=1$, and in the absence of absorption, $T_{12}=T_{21}^*$, $T_{11}=T_{22}^*$. The transfer matrix approach was implemented in C++ programming language.
